# Treatment of Agitation With Lorazepam in Clinical Practice: A Systematic Review

**DOI:** 10.3389/fpsyt.2021.628965

**Published:** 2021-02-22

**Authors:** Mario Amore, Mariella D'Andrea, Andrea Fagiolini

**Affiliations:** ^1^Section of Psychiatry, Department of Neuroscience, Ophthalmology, Genetics and Infant-Maternal Science, University of Genoa, Genoa, Italy; ^2^Istituto di Ricovero e Cura a Carattere Scientifico Ospedale Policlinico San Martino, Genoa, Italy; ^3^Medical Department, Pfizer Italia, Rome, Italy; ^4^Department of Molecular and Developmental Medicine, University of Siena, Siena, Italy

**Keywords:** lorazepam, agitation, systematic review, benzodiazepine, clinical trial

## Abstract

Acute agitation is a frequent occurrence in both inpatient and outpatient psychiatric settings, and the use of medication to calm a patient may be warranted to mitigate the situation. Lorazepam is a benzodiazepine that is widely used for management of acute agitation. Despite its widespread use, there is remarkably little clinical evidence for the benefits of lorazepam in acute agitation. We performed a systematic review with focus on lorazepam, including all randomized clinical trials on lorazepam in mental and behavioral disorders, excluding studies on dementia and pediatric patients and in mixed conditions. A total of 11 studies met inclusion criteria, and all were in patients with mental and behavioral disorders. Most trials generally found improvements across a variety of outcomes related to agitation, although there was some disparity if specific outcomes were considered. In the five studies with haloperidol, the combination of lorazepam and haloperidol was superior to either agent alone, but with no differences between monotherapy with the individual agents. In the study comparing lorazepam to olanzapine, olanzapine was superior to lorazepam, and both were superior to placebo. As expected, the safety of lorazepam among the different studies was consistent with its well-characterized profile with dizziness, sedation, and somnolence being the most common adverse events. Based on this structured review, lorazepam can be considered to be a clinically effective means of treating the acutely agitated patient.

## Introduction

Agitation in patients with psychiatric conditions is a frequent occurrence and an issue of substantial clinical relevance in psychiatry in emergency settings and in both inpatient and outpatient psychiatric settings (1). Agitation is associated with many psychiatric conditions in addition to substance use and/or intoxication and conditions involving the central nervous system (e.g., Parkinson's disease, Alzheimer's disease, dementia, etc.) and brain trauma (1). The key features generally recognized in patients with agitation include restlessness with excessive or semi-purposeful motor activity, irritability, and augmented responses to internal and external stimuli, together with an unstable clinical course (2). The DSM-5 defines agitation as excessive motor activity associated with a feeling of inner tension, which is frequently accompanied by non-productive, repetitious of behaviors like pacing, fidgeting, wringing of the hands, pulling of clothes, and inability to sit still (3). Progression of agitation can also lead to violence and aggressive behavior (4).

When agitation is severe it can be accompanied by complete lack of behavioral control where the threat of damage to property, assault to others, and self-inflicted injury are of immediate concern (5). In such a clinical condition, the use of medication to calm down the patient may be warranted to mitigate the overall situation through immediate administration of medication, with or without the patient's consent. Given the clinical relevance and impact of agitation, prompt evaluation of causative factors and immediate management are crucial, since this may the healthcare provider to gain control over potentially hazardous behaviors (1). The overarching goal of medication in the management of acute agitation is to rapidly calm the patient without oversedation (6). Assessment of the causes of agitation allow the clinician to choose the most appropriate management strategy. When agitation is due to delirium or another physical condition such as brain trauma, the underlying organic causes should be addressed; if agitation is related to an underlying mental condition such as schizophrenia or bipolar disorder, antipsychotics and/or benzodiazepines are normally considered (7). In fact, intramuscular injections of typical antipsychotics and benzodiazepines, either alone or in combination, have remained the mainstay of treatment for decades, although the use of intramuscular atypical antipsychotics has gained widespread acceptance (5). The intramuscular formulations of atypical antipsychotics indicated for acute agitation include ziprasidone, olanzapine, and aripiprazole (8). Although no direct comparative studies with these intramuscular agents have been carried out, it is generally held that their efficacy is comparable for acute agitation and similar to intramuscular haloperidol (9). While a wide choice of treatments are available, current recommendations on agitation in psychiatry are not univocal.

Lorazepam is a widely used benzodiazepine that has been available for more than 40 years (10). Lorazepam is frequently used as the sedative and anxiolytic of choice in inpatient settings due to its rapid (1–3 min) onset of action when administered intravenously, and a relatively good safety profile. Lorazepam is often used for episodes of acute agitation. Despite its widespread use, there is surprisingly scarce clinical evidence for the benefits of lorazepam (and other benzodiazepines) in acute agitation. For example, a recent Cochrane review concluded that the evidence for the use of benzodiazepines is not high, and that the advantage of adding a benzodiazepine to other drugs is not entirely clear, also in light of potential additive adverse effects (11). Others have concluded that a first-generation antipsychotic together with lorazepam or monotherapy with lorazepam or a second-generation antipsychotic are effective therapeutic options for acute agitation (4, 12).

To shed more light in the use of benzodiazepines in managing patients with acute agitation, we performed a systematic review with particular focus on lorazepam. For the purposes of this review, in order to focus on a somewhat homogeneous population, we included all randomized clinical trials on lorazepam in mental and behavioral disorders, excluding studies on dementia and pediatric patients and in mixed conditions such as cancer and AIDS. For the purposes of the present review, attempt was made to distinguish between agitation, violence, or aggressive behavior.

## Materials and Methods

This systematic review was conducted following the Preferred Reporting Items for Systematic Reviews and Meta-Analyses (PRISMA) guidelines (13, 14). In April 2020, we conducted a systematic literature search in the PubMed/Medline database and in the Cochrane Library for papers reporting data on the use of lorazepam in agitated patients. The following search string was used (lorazepam OR Ativan OR Orfidal OR Témesta OR Tolid OR Donix OR Duralozam OR Durazolam OR Idalprem OR Laubeel OR Lorazep OR Lorazepam-Neuraxpharm OR Lorazepam-Ratiopharm OR Novo-Lorazem OR Novolorazem OR Nu-Loraz OR Sedicepan OR Sinestron OR wy-4036 OR wy4036 OR Apo-Lorazepam OR Somagerol OR Temesta) AND (agitation OR aggression OR violence OR delirium OR confusion). The same combination of terms was used in the exploration of the Cochrane Library database. No restrictions on language or type of study were applied, and a meta-analysis was not planned.

Two members of the review team retrieved and evaluated independently the potentially relevant articles, and checked the reference list of all reviews and papers of interest to obtain other pertinent publications. An independent search in Google Scholar was also performed, in order to identify other papers that had been missed. Conference abstracts were evaluated, but none reported sufficient data for inclusion. Unpublished studies were excluded.

No studies were excluded a priori for weakness of design or data quality. Publications identified were included if the following criteria were met: randomized controlled trials (RCT) on lorazepam use, as a single agent or in combination with other drugs, reporting quantitative information on efficacy and/or safety of lorazepam in agitated patients. Studies on both oral and intramuscular lorazepam use were included. On the other hand, publications identified were excluded according to the following criteria: studies not specifically focused on patients with agitation (e.g., studies of patients with alcohol withdrawal syndrome, unless they examined only patients with incipient delirium; studies of mixed psychotic conditions that included also non-agitated patients; etc.); studies not focused on lorazepam (e.g., those that reported use of lorazepam as rescue medication); duplicate publications that did not contain additional data; case-series; case-reports. Studies on agitation associated with agitation/delirium in AIDS, cancer, brain injury, dementia, and alcoholism were excluded, as were those on pediatric patients. Discrepancies between members of the review team were discussed and resolved.

Two members of the review team examined all the publications that had been selected for inclusion, evaluated risk of bias, and abstracted the following information in a standard format: country; study period; study design (RCT or other); number of patients enrolled; underlying condition and study setting; intervention(s) characteristics, including drugs, dose and type of administration (oral, intramuscular, intravenous); efficacy/effectiveness outcomes reported; safety outcomes reported. We extracted results on any available efficacy outcome presented in each publication, such as the proportion of patients tranquil or asleep at a given timepoint, the Brief Psychiatric Rating Scale (BPRS), the Clinical Global Impression (CGI) score, and the Excited Component of the Positive and Negative Syndrome Scale (PANSS-EC), by reporting data on the percentage of patients achieving the efficacy target or the median/mean score of each outcome in the lorazepam and comparison groups; similarly, we extracted information on several safety outcomes, such as the proportion of any adverse event, of severe adverse events and of extrapyramidal syndrome (EPS) symptoms, in each group. When appropriate and available, findings for the comparison of efficacy and safety between groups (in terms of *p*-values or relative risks) were also abstracted. Differences between data extracted by the members of the review team were further checked on the original articles, and resolved.

## Results

**Figure 1:**
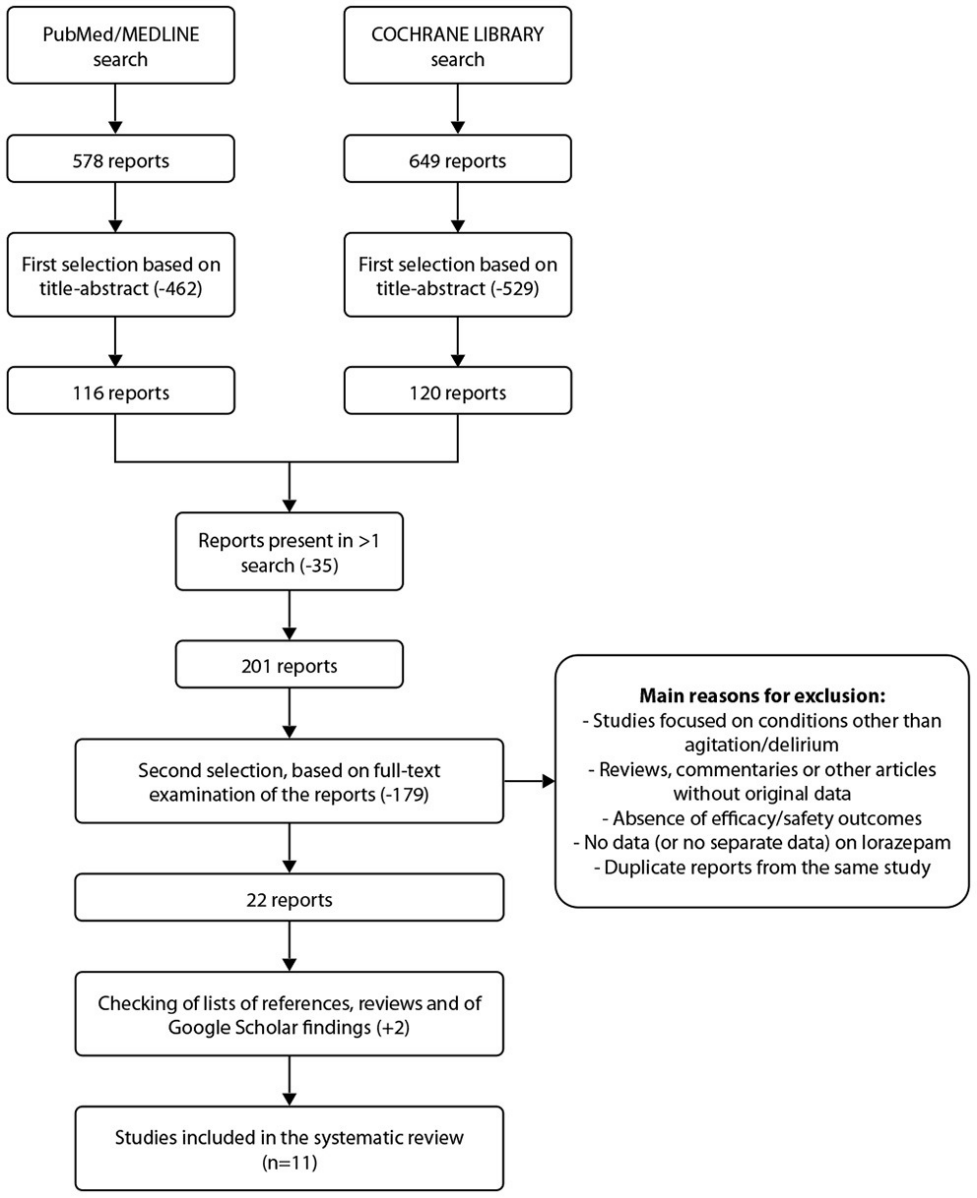
Flowchart for search and selection of studies on lorazepam in agitated patients.

Figure 1 shows the process of search and selection of publications for the present systematic review. A total of 578 publications were retrieved from PubMed and 649 from the Cochrane library. Following removal of duplicates, 201 publications remained and were subjected to full text analysis. After final review, based on inclusion criteria, 11 studies were included in the present analysis. The main characteristics of the trials included are shown in Supplementary Table 1. Of the 11 trials, 3 studied lorazepam only in combination with other medications, i.e., not as monotherapy, and all were in patients with mental and behavioral disorders. The main efficacy results of randomized controlled trials on lorazepam for treatment of patients with agitation are summarized in Supplementary Table 2.

### Efficacy

In these 11 studies, a wide variety of outcome measures were used. The most commonly used was CGI or CGI subscales (*n* = 7) followed by the PANNS (*n* = 5). Three studies each used the BPRS, ACES, and VAS agitation.

Haloperidol was the comparator in 5 studies. With the exception of the trial by Salzman et al. (15), in general, significant differences were seen favoring lorazepam over haloperidol, and the combination of lorazepam + haloperidol was superior to lorazepam alone. However, while broadly speaking the differences seen favored lorazepam, the differences were not consistent across trials. For example, Garza-Trevino et al. reported no differences in VAS agitation at 60 min (16), while Bieniek et al. reported significant differences in VAS agitation at 1 h (17). The trial by Battaglia et al. found no differences between groups in CGI at 3 h (18), in contrast to that by Foster et al. who reported significant differences in favor of lorazepam at 1–3 h (19).

The study by Battaglia et al. reported that olanzapine was significantly more effective than placebo considering changes in PANNS-EC at 2 h; the p values for lorazepam vs. placebo were not reported (20). The trial by Alexander et al. compared lorazepam to the combination of haloperidol and promethazine, reporting that the combination was more effective at 2 h than lorazepam with a faster onset of action. Zimbroff et al. compared aripiprazole to lorazepam and placebo, reporting that lorazepam was significantly more effective than placebo for all outcome measures, but with no difference between lorazepam and the antipsychotic (21).

Three studies compared lorazepam in combination with other agents: (i) thiothixene and lorazepam vs. haloperidol and phenobarbital sodium (16); intramuscular (IM) administration of lorazepam and haloperidol vs. oral risperidone + lorazepam (22); IM olanzapine vs. haloperidol + lorazepam (23). Using these combinations, all three studies documented significant efficacy for both study arms, but with no significant differences between groups for any outcome measure.

Supplementary Table 3 reports an evaluation of the quality and of the risk of bias in each study included in this systematic review.

### Safety

Supplementary Table 4 summarizes the main findings on the safety of lorazepam and comparators in the treatment of patients with agitation. The safety profile of lorazepam, alone or in combination, was as expected. Dizziness was reported in ≥10% of patients in 3 trials; sedation/somnolence was documented in about 10% of patients in three trials as well.

## Discussion

The present systematic review on the use of lorazepam in acute agitation highlights that there is a paucity of randomized trials. Despite this, most trials generally found improvements across a variety of outcomes related to agitation, even if there was some disparity among different studies when considering specific outcomes. As expected, the safety of lorazepam among the different studies was consistent with its well-characterized profile. Among the studies included in the present analysis, the most frequently used comparators were haloperidol and a second-generation antipsychotic, as monotherapy or in various combinations, which is consistent for the most part with routine practice. The studies were highly heterogeneous, especially regarding treatment arms, doses, and outcome measures, rendering meta-analysis impossible. Indeed, the differences among studies even make overall qualitative evaluation difficult.

In general, in the studies with haloperidol, the combination of lorazepam and haloperidol was superior to either agent alone, with significant differences favoring lorazepam over haloperidol (15–19). In the study comparing lorazepam to olanzapine, olanzapine was superior to lorazepam, and both were superior to placebo (20). In the three studies comparing combinations of agents, interpretation is rendered difficult by the lack of monotherapy groups (16, 22, 23), and so the effects of lorazepam or other comparators cannot be directly interpreted.

Qualitative analysis of the safety profile of lorazepam from the different studies revealed no new safety issues, with dizziness, sedation and somnolence being common among the trials that listed specific adverse events. Haloperidol, but not lorazepam apart from isolated case reports (24), is known to be associated with alterations in QTc (25). This was reported to be of concern for patients with torsade de pointes, but not in the great majority of patients. Case reports with QTc prolongation have also been documented (26), but the event does not seem to be common and QTc prolongation is not reported in the Summary of Prescribing Characteristics. Also, unlike many antipsychotics, routine monitoring of the QT duration by electrocardiography prior to treatment is not recommended for lorazepam (27).

According to the recent expert consensus of treatment of psychomotor agitation, non-pharmacological approaches should be attempted first, with the involvement of the patient in therapeutic decisions as much as possible (1). In the event that these methods are not adequate, pharmacological treatment may be considered in order to rapidly calm an agitated patient. As mentioned, over-sedation should be avoided, and oral medications are preferred. However, in some patients, escalation to IM medication is needed. Rapid onset and the reliability are considered to be the most important factors to consider when choosing a route of administration. Lorazepam is often an anxiolytic of choice, given its rapid onset of action (10).

This systematic review was carried out to evaluate the efficacy and safety of lorazepam for acute agitation and thus better understand its suitability for use in the acute setting. A total of 11 randomized clinical trials were included. Our study has several limitations. First, the heterogeneity of trials from multiple points of view hindered additional analyses. Second, among the studies included, there were little or no available on the clinical implications of rapidity of onset of efficacy, other than the first time point in the respective analysis, or relevant information on use of restraint or seclusion or length of stay. Insightful inter-study comparison of clinical data within the context of this review was further confounded by differences in study design. The trials differed in measures used to assess agitation; many used multiple outcomes measures, and some used only one, with no commonly used measure. Although the scales utilized may be a valid means to measure agitation, the use of differ but outcome measures make comparisons problematic. The degree of agitation among the different studies may also vary. Lastly, it is clear that for inclusion in clinical trials patients have to be unwell enough to warrant invasive intervention, but well enough to give informed consent so that some patients are excluded from inclusion. Based on our analysis, lorazepam seems to be superior to placebo (but not to other treatments) in management of agitation.

The optimal management strategy patients with agitation should begin with quick assessment of possible medical conditions, and non-pharmacological intervention (1). When these methods fail, use of restraint and medications can be considered. The physician must consider the time of onset and risk for adverse events when choosing a medication. The most widely used agents are typical and atypical antipsychotics, benzodiazepines, and combination therapies (5). In a study in Belgium, for example, the preferred medication classes were antipsychotics (59%) and benzodiazepines (41%); among the latter, lorazepam was the preferred drug (28).

Based on this structured review, and despite its limitations, the present analysis reinforces that lorazepam can be considered to be a clinically effective means of treating the acutely agitated patient. However, the choice of drug(s) for rapid tranquilization remains a matter of clinical judgement until additional well-designed studies with larger cohorts of patients are carried out in settings that are more reflective of routine practice.

## Data Availability Statement

The original contributions generated for the study are included in the article/Supplementary Material, further inquiries can be directed to the corresponding author/s.

## Author Contributions

MA, MD'A, and AF contributed equally to the conception, design, and execution of the study, to the drafting and revisions of the manuscript, and read and approved the submitted version. All authors contributed to the article and approved the submitted version.

## Conflict of Interest

MA is /has been a consultant and/or a speaker and/or has received research grants from Angelini, FB-Health, Janssen, Lundbeck, Otsuka, Pfizer, Recordati, and Glaxo. AF is/has been a consultant and/or a speaker and/or has received research grants from Allergan, Angelini, Aspen, Boehringer Ingelheim, Daiichi Sankyo Brasil Farmacêutica, Doc Generici, FB-Health, Italfarmaco, Janssen, Lundbeck, Mylan, Otsuka, Pfizer, Recordati, Sanofi Aventis, Sunovion, and Vifor. MD'A is a Pfizer employee.
