# Supplementary material for: Treatment of Agitation With Lorazepam in Clinical Practice: A Systematic Review
Source: Front Psychiatry. 2021 Feb 22;12:628965. doi: 10.3389/fpsyt.2021.628965 (PMC7937895; doi:10.3389/fpsyt.2021.628965)
Supplement: Supplementary file 1 [file Table_1.docx]

### **Supplementary Table 1.** Main characteristics of randomized clinical trials on the efficacy and safety of lorazepam in treatment of patients with agitation.

| Reference | Country, Period | Study design | Study size | Condition, Setting | | Intervention(s) / Comparator(s) | | | | Efficacy outcomes | | | | Safety outcomes |
| --- | --- | --- | --- | --- | --- | --- | --- | --- | --- | --- | --- | --- | --- | --- |
| Mental and behavioral disorders | | | | |  | | |  | |  | |  | |  |
| *Monotherapy or combination therapy* | | | | |  | | |  | |  | |  | |  |
|  |  |  |  |  | |  | | | |  | | | |  |
| Garza-Trevino et al., 1989  Study 2 | USA, NR | RCT | 68 pts randomized to 3 groups | Psychotic agitation, Psychiatric hospital | | 1) IM lorazepam (4 mg)  2) IM haloperidol (5 mg)  3) IM lorazepam (4 mg) + haloperidol (5 mg) | | | | Reduction of VAS agitation to 20 or less at 30, 60 and >60 m | | | | Not reported |
|  |  |  |  |  | |  | | | |  | | | |  |
| Salzman et al., 1991 | USA,  NR | RCT | 60 pts divided in 2 groups | Aggression, agitation and assaultive behavior; Psychiatric Emergency | | 1) IM lorazepam (2 mg)  2) IM haloperidol (5 mg) | | | | - BPRS  - CGI-S  - Tranquil or asleep at 60 min  - OAS | | | | - Any AE  - EPS symptoms |
|  |  |  |  |  | |  | | | |  | | | |  |
| Battaglia et al., 1997 | USA,  NR | RCT | 98 pts randomized to 3 groups | Psychosis and behavioral dyscontrol, emergency department | | 1) IM lorazepam (2 mg)  2) IM haloperidol (5 mg)  3) IM lorazepam (2 mg) + haloperidol (5 mg) | | | | 12-hour evaluation of:  - Modified BPRS  - ABS  - CGI | | | | CNS AE |
|  |  |  |  |  | |  | | | |  | | | |  |
| Foster et al., 1997 | USA,  NR | RCT | 37 pts randomized to 2 groups | Psychotic agitation; Psychiatric Emergency service | | 1) Lorazepam (2 mg)  2) Haloperidol (5 mg) | | | | Change at 1, 2, 3 and 4 hours in:  - BPRS  - CGI | | | | - EPS symptoms |
|  |  |  |  |  | |  | | | |  | | | |  |
| Bieniek et al., 1998 | USA,  NR | RCT | 20 pts randomized to 2 groups | Psychiatric disorders, Psychiatric emergency | | 1) IM lorazepam (2 mg)  2) IM lorazepam (2 mg) + haloperidol (5 mg) | | | | Change at 30, 60, 120 and 180 minutes in:  - OAS  - VAS agitation and hostility  - CGI-S  - Sedation | | | | Any AE |
|  |  |  |  |  | |  | | | |  | | | |  |
| Battaglia et al., 2003  Study 2 | USA & Romania, NR | RCT | 201 pts randomized to 3 groups | Bipolar disorder; inpatients | | 1) IM olanzapine (10 mg)  2) IM lorazepam (2 mg)  3) IM placebo | | | | Change at 2 h in PANSS-EC score in non-oversedated pts (ACES<7) | | | | - Incidence of oversedation (ACES>7) at 24 h  - Sedation AE at 24 h |
|  |  |  |  |  | |  | | | |  | | | |  |
| Alexander et al., 2004 | India, 2002 | Pragmatic RCT | 221 pts enrolled;  200 pts randomized to 2 groups | Psychiatric disorders, Psychiatric emergency | | 1) IM haloperidol (10 mg) and promethazine (25 or 50 mg)  2) IM lorazepam (4 mg) | | | | At 15, 30, 60, 120 and 240 minutes:  - Tranquil/asleep  - Asleep  - Improved  - In restraints  - Use of other drugs  - CGI  - Time to tranquil  - Time to sleep | | | | - Any AE within 240 minutes  - Serious AE at 2 weeks |
|  |  |  |  |  | |  | | | |  | | | |  |
| Zimbroff et al., 2007 | USA,  NR | RCT | 301 pts randomized to 4 groups | Agitation in pts with bipolar I disorder; inpatients | | 1) IM aripiprazole (9.75 mg)  2) IM aripiprazole (15 mg)  3) IM lorazepam (2 mg)  4) IM placebo | | | | Change at 2 h in:  - PANSS-EC score  - PANSS-EC response rate  - CGI-I  - CGI-S  - ACES  - CABS  - YMRS  - SAS  - BARS | | | | - Any AE over 24 h |
| *Combination therapy only* | | | | | | |  | |  | |  | |  |  |
|  |  |  |  |  | |  | | | |  | | | |  |
| Garza-Trevino et al., 1989  Study 3 | USA, NR | RCT | 53 pts randomized to 2 groups | Psychotic agitation, Psychiatric hospital | | 1) thiothixene (5 mg) + lorazepam (4 mg)  2) phenobarbital (130 mg) + haloperidol (5 mg) | | | | Reduction of VAS agitation to 20 or less at 30, 60 and >60 m | | | | Not reported |
|  |  |  |  |  | |  | | | |  | | | |  |
| Currier et al., 2004 | USA,  2001 | RCT | 181 pts screened, 162 randomized to 2 groups | Psychotic agitation, emergency department or inpatients | | 1) oral risperidone (2 mg) + oral lorazepam (2 mg)  2) IM lorazepam (2 mg) + IM haloperidol (5 mg) | | | | Change at 0.5, 1, 2, 3, 6 and 24 h in:  - 5-item PANSS  - Total PANSS  - CGI-S  - OAS | | | | - Any AE  - Movement disorders  - BAS  - Severity of parkinsonism |
|  |  |  |  |  | |  | | | |  | | | |  |
| Huang et al. 2015 | Taiwan, 2006-09 | RCT | 70 pts enrolled, 67 randomized to 2 groups | Agitated pts with schizophrenia or schizoaffective disorder, inpatients | | 1) IM olanzapine (10 mg)  2) IM lorazepam (2 mg) + IM haloperidol (5 mg) | | | | Change at 15, 30, 60 and 120 minutes in:  - PANSS-EC  - ACES  - CGI-S | | | | - Any AE in 24 h  - SAS  - BARS |

ABS: Agitated Behavior Scale; ACES: Agitation-Calmness Evaluation Scale; AE: Adverse events; BARS: Barnes Akathisia Rating Scale; BPRS: Brief Psychiatric Rating Scale; CABS: Corrigan Agitated Behavior Scale; CGI-I: Clinical Global Impression - Improvement; CGI-S: Clinical Global Impression - Severity; CMAI: Cohen-Mansfield Agitation Inventory; CNS: central nervous system; CT: controlled trial; DRS: delirium rating scale; EPS: Extrapyramidal syndrome; IM: intramuscular; IV: intra-venous; MDAS: Memorial Delirium Assessment Score; MMSE: mini-mental state examination; NR: not reported; OAS: Overt Aggression Scale; PANSS-EC: Positive and Negative Syndrome Scale – Excited Component; RASS: Richmond Agitation-Sedation Scale; RCT: randomized controlled trial; SAS: Simpson-Angus Scale; VAS: Visual Analog Scale; YMRS: Young Mania Rating Scale

### **Supplementary Table 2.** Main efficacy results of randomized controlled trials on lorazepam for treatment of patients with agitation.

| Reference | N,  lor | N,  comp 1 | N,  comp 2 | Endpoints | Efficacy,  lor | Efficacy,  comp 1 | Efficacy,  comp 2 | Main result for comparison |
| --- | --- | --- | --- | --- | --- | --- | --- | --- |
| Mental and behavioral disorders | | | | |  |  |  |  |
| *Monotherapy or combination therapy* | | | | |  |  |  |  |
| Garza-Trevino et al., 1989  Study 2 | 23 | 21  (hal) | 24  (hal+lor) | VAS agitation ≤20 (at 30 m)  VAS agitation ≤20 (at >60 m) | 39.1%  100% | 33.3%  100% | 75.0%  100% | L+h superior to lor and hal alone  No difference |
|  |  |  |  |  |  |  |  |  |
| Salzman et al., 1991 | 30 | 30  (hal) | - | Tranquil or asleep (1 h)  Asleep (1 h) | 70.0%  30.0% | 73.3%  26.7% |  | No difference between groups  No difference between groups |
|  |  |  |  |  |  |  |  |  |
| Battaglia et al., 1997 | 31 | 35  (hal 5) | 32  (lor+hal) | ABS (over 12 h)  Modified BPRS (over 12 h)  Asleep (3 h)  CGI improvement (3h) | Improved  Improved  65%  ≈90% | Improved  Improved  32%  ≈90% | Improved  Improved  61%  ≈70% | L+h superior to lor; no other differences  L+h superior to lor and hal at hour 2 & 3  Lor superior to hal; L+h superior to hal  No differences between groups |
|  |  |  |  |  |  |  |  |  |
| Foster et al., 1997 | 17 | 20  (hal) | - | BPRS (1 h)  BPRS (2 h)  BPRS (3 h)  BPRS (4 h)  CGI (1 h)  CGI (2 h)  CGI (3 h)  CGI (4 h) | 52.1 (11.2)  45.3 (10.0)  40.4 (11.3)  38.6 (9.8)  4.9 (0.7)  4.2 (0.7)  3.9 (1.0)  3.5 (1.2) | 55.4 (11.7)  49.4 (10.7)  45.5 (13.5)  40.3 (14.4)  5.5 (0.6)  5.0 (1.0)  4.9 (1.1)  4.1 (1.0) |  | Efficacy in both groups, no difference  Efficacy in both groups, no difference  Efficacy in both groups, no difference  Efficacy in both groups, no difference  Efficacy in both groups, p<0.05 favor lor  Efficacy in both groups, p<0.05 favor lor  Efficacy in both groups, p<0.05 favor lor  Efficacy in both groups, no difference |
|  |  |  |  |  |  |  |  |  |
| Bieniek et al., 1998 | 11 | 9  (lor+hal) | - | OAS improvement (1 h)  VAS agitation improvement (1 h)  CGI-S improvement (1 h)  VAS Sedation (1 h) | 55%  27%  55%  +30 in VAS | 100%  78%  33%  +45 in VAS |  | L+h superior to lor  L+h superior to lor  No difference  No difference |
|  |  |  |  |  |  |  |  |  |
|  |  |  |  |  |  |  |  |  |
| Battaglia et al., 2003  Study 2: Same study as Meehan 2001 | 44 | 60  (ola) | 47  (pla) | PANSS-EC change (2 h), excluding oversedated patients (ACES=7, 8 or 9) | -5.8 (4.6) | -8.0 (5.0) | -4.5 (4.6) | Ola vs lor: p=0.018; Lor vs pla: p=NR |
|  |  |  |  |  |  |  |  |  |
| Alexander et al., 2004 | 100 | 100  (hal+pro) |  | Tranquil/asleep (240 m)  Asleep (240 m)  Clinically improved (240 m)  In restraints (240 m)  Use of other drugs (240 m)  CGI score (240 m)  Time to tranquillization (m)  Time to sleep (m) | 96%  45%  86%  11%  9%  1.91 (0.67)  47.8 (46.7)  80.6 (64.3) | 96%  76%  87%  9%  8%  1.82 (0.99)  29.7 (35.6)  37.4 (42.9) |  | RR=1.0 (0.9-1.1) comp vs lor  RR=1.7 (1.3-2.2) comp vs lor  RR=1.0 (0.9-1.1) comp vs lor  RR=0.8 (0.3-1.9) comp vs lor  RR=0.9 (0.4-2.2) comp vs lor  p=0.005 favor comp (RM over time)  p<0.001 favor comp  p<0.001 favor comp |
|  |  |  |  |  |  |  |  |  |
| Zimbroff et al., 2007 | 68 | 75  (ari 9.75) | 73  (pla) | PANSS-EC score change (2 h)  PANSS-EC response rate (2 h)  CGI-I (2 h)  CGI-S change (2h)  ACES change (2h)  CABS change (2 h)  YMRS change (2h) | -9.6  69%  2.1  -1.6  2.3  -10.4  -10.8 | -8.7  69%  2.2  -1.5  1.9  -9.6  -11.4 | -5.8  37%  3.1  -0.9  1.0  -6.4  -7.0 | Lor vs. pla: <0.001; Lor vs. ari: no diff  Lor vs. pla: <0.01; Lor vs. ari: no diff  Lor vs. pla: <0.01; Lor vs. ari: no diff  Lor vs. pla: <0.01; Lor vs. ari: no diff  Lor vs. pla: <0.01; Lor vs. ari: no diff  Lor vs. pla: <0.01; Lor vs. ari: no diff  Lor vs. pla: <0.05; Lor vs. ari: no diff |
| *Combination therapy only* | | | | |  |  |  |  |
| Garza-Trevino et al., 1989  Study 3 | 26 (lor+th) | 27 (hal+ph) |  | VAS agitation ≤20 (at 30 m)  VAS agitation ≤20 (at >60 m) | 57.7%  96.2% | 55.6%  88.9% |  | No difference  No difference |
|  |  |  |  |  |  |  |  |  |
| Currier et al., 2004 | 83  (oral ris+lor) | 79  (IM hal+lor) |  | 5-item PANSS change  Total PANSS change  CGI-S marked to severe change  OAS change (2 h) | -8.0 (0.4)  -19.0 (1.6)  -29%  -3.4 (0.1) | -8.4 (0.5)  -18.4 (1.9)  -32%  -3.5 (0.1) |  | Efficacy in both groups, no difference  Efficacy in both groups, no difference  No test reported  Efficacy in both groups, no difference |
|  |  |  |  |  |  |  |  |  |
| Huang et al., 2015 | 30 (hal+lor) | 37  (ola) |  | PANSS-EC change (2 h)  PANSS-EC change (24 h)  ACES change (2 h)  ACES change (24 h)  CGI-S change (24 h) | -9.9 (5.6)  -8.1 (3.1)  2.2 (1.7)  1.3 (1.0)  -0.8 (0.7) | -10.2 (6.5)  -9.2 (5.4)  2.1 (1.7)  1.2 (0.9)  -0.8 (0.6) |  | Efficacy in both groups, no difference  Efficacy in both groups, no difference  Efficacy in both groups, no difference  Efficacy in both groups, no difference  Efficacy in both groups, no difference |

ABS: Agitated Behavior Scale; ACES: Agitation-Calmness Evaluation Scale; BPRS: Brief Psychiatric Rating Scale; CABS: Corrigan Agitated Behavior Scale; CGI-I: Clinical Global Impression - Improvement; CGI-S: Clinical Global Impression - Severity; CMAI: Cohen-Mansfield Agitation Inventory; DRS: delirium rating scale; IM: intramuscular; MDAS: Memorial Delirium Assessment Score; MMSE: mini-mental state examination; NR: not reported; OAS: Overt Aggression Scale; PANSS-EC: Positive and Negative Syndrome Scale – Excited Component; RASS: Richmond Agitation-Sedation Scale; VAS: Visual Analog Scale; YMRS: Young Mania Rating Scale.

**Supplementary Table 3.** Quality measures of randomized controlled trials included in the systematic review.

| **Author, year** | **Blinding** | **Risk of selection bias ^a^** | **Risk of data incompleteness** | **Risk of selective reporting** |
| --- | --- | --- | --- | --- |
| Garza-Trevino et al., 1989 | Not blinded | Unclear | Low | Low |
| Salzman et al., 1991 | Double-blind | Unclear | Unclear | Low |
| Battaglia et al., 1997 | Double-blind | Low | Unclear | Low |
| Foster et al., 1997 | Double-blind | Low | Low | Unclear |
| Bieniek et al., 1998 | Double-blind | Low | Low | Yes |
| Alexander et al., 2004 | Blinded only until treatment assignment | Low | Low | Low |
| Currier et al., 2004 | Blinded only to raters | Low | Yes | Low |
| Zimbroff et al., 2007 | Double-blind | Unclear | Low | Low |
| Huang et al. 2015 | Not blinded | Low | Low | Low |

^a^ Evaluated according to the presence/absence of an allocation concealment strategy and to random number generation in each study.

### **Supplementary Table 4.** Main safety results of studies of lorazepam in the treatment of patients with agitation.

| Reference | N,  lor | N,  comp 1 | N,  comp 2 | Endpoint | Safety,  lor | Safety,  comp 1 | Safety, comp 2 | Main result for comparison |
| --- | --- | --- | --- | --- | --- | --- | --- | --- |
| Mental and behavioral disorders | | | | |  |  |  |  |
| *Monotherapy or combination therapy* | | | | |  |  |  |  |
|  | | | | |  |  |  |  |
| Garza-Trevino et al., 1989  Study 2 | 23 | 21  (hal) | 24  (hal+lor) | Not reported | NR | NR |  | - |
|  |  |  |  |  |  |  |  |  |
| Salzman et al., 1991 | 30 | 30  (hal) |  | EPS symptoms | 3.3% | 50.0% |  | Lor vs. hal: p<0.001 |
|  |  |  |  |  |  |  |  |  |
| Battaglia et al., 1997 | 31 | 35  (hal 5) | 32  (lor+hal) | EPS symptoms  Ataxia  Dizziness  Dry mouth  Speech disorders | 3%  6%  10%  16%  6% | 20%  3%  9%  9%  11% | 6%  9%  6%  9%  9% | No differences between groups  No differences between groups  No differences between groups  No differences between groups  No differences between groups |
|  |  |  |  |  |  |  |  |  |
| Foster et al., 1997 | 17 | 20  (hal) |  | EPS symptoms (by 4 h)  Extreme sedation | 0.0%  17.6% | 0.0%  10.0% |  | No differences between groups  No differences between groups |
|  |  |  |  |  |  |  |  |  |
| Bieniek et al., 1998 | 11 | 9  (lor+hal) |  | Any AE | 0% | 0% |  | No differences between groups |
|  |  |  |  |  |  |  |  |  |
| Battaglia et al., 2003  Study 2 - Same study as Meehan 2001 | 51 | 99  (ola) | 51  (pla) | Oversedation (pts ACES ≥8) | 3.9% | 6.1% | 0.0% | No difference vs ola; no difference vs pla |
|  |  |  |  |  |  |  |  |  |
| Alexander et al., 2004 | 100 | 100  (hal+pro) |  | Any AE (240 m)  Serious AE (at 2 wk) | 0%  0% | 2%  0% |  | NR  RR=1.0 (0.9-1.1) comp vs lor |
|  |  |  |  |  |  |  |  |  |
| Zimbroff et al., 2007 | 68 | 75  (ari 9.75) | 73  (pla) | Oversedation (ACES ≥8)  Headache  Insomnia  Dizziness  Nausea  Somnolence  Sedation  Vomiting  EPS symptoms  SAS change (2h)  BARS change (2h) | 6.8%  4.4%  1.5%  10.1%  0.0%  7.3%  11.6%  0.0%  0.0%  -0.5  -0.4 | 6.7%  14.7%  10.7%  2.7%  10.7%  8.0%  4.0%  4.0%  4.0%  -0.6  -0.4 | 19.1%  12.5%  8.3%  5.6%  5.6%  5.6%  1.4%  1.4%  1.4%  -0.5  -0.4 | Increased (p<0.05) for lor vs. ari and pla  NR  NR  NR  NR  NR  NR  NR  NR  No difference between groups  No difference between groups |
| *Combination therapy only* |  |  |  |  |  |  |  |  |
|  |  |  |  |  |  |  |  |  |
| Garza-Trevino et al., 1989  Study 3 | 26 (lor+th) | 27 (hal+ph) |  | Not reported | NR | NR |  | - |
|  |  |  |  |  |  |  |  |  |
|  |  |  |  |  |  |  |  |  |
| Currier et al., 2004 | 83  (oral ris+lor) | 79  (IM hal+lor) |  | Any treatment-related AE  Headache  Hyperkinesia  Somnolence  Agitation  Movement disorders  BARS change  SAS score change | 24%  4.8%  1.2%  13.3%  4.8%  4.8%  -0.6  -0.3 | 25%  6.3%  5.1%  12.7%  5.1%  10.2%  -0.3  0.1 |  | No differences between groups  No differences between groups  No differences between groups  No differences between groups  No differences between groups  No differences between groups  No differences between groups  No differences between groups |
|  |  |  |  |  |  |  |  |  |
| Huang et al., 2015 | 30 (hal+lor) | 37  (ola) |  | SAS change  BARS change  Any AE | -0.6  -0.6  33.3% | -0.4  -0.8  24.3% |  | No differences between groups  No differences between groups  No differences between groups |

ACES: Agitation-Calmness Evaluation Scale; AE: Adverse events; BARS: Barnes Akathisia Rating Scale; CNS: central nervous system; EPS: Extrapyramidal syndrome; IM: intramuscular; NR: not reported; NS: not significant; RCT: randomized controlled trial; SAS: Simpson-Angus Scale.
